# I-scan optical enhancement for the *in vivo* prediction of diminutive colorectal polyp histology: Results from a prospective three-phased multicentre trial

**DOI:** 10.1371/journal.pone.0197520

**Published:** 2018-05-16

**Authors:** Entcho Klenske, Steffen Zopf, Clemens Neufert, Andreas Nägel, Jürgen Siebler, Jürgen Gschossmann, Steffen Mühldorfer, Lukas Pfeifer, Sarah Fischer, Francesco Vitali, Marietta Iacucci, Subrata Ghosh, Michelle G. Rath, Peter Klare, Gian E. Tontini, Markus F. Neurath, Timo Rath

**Affiliations:** 1 Department of Medicine I, Division of Gastroenterology, Ludwig Demling Endoscopy Center of Excellence, University Hospital of Erlangen, Erlangen, Germany; 2 Department of Gastroenterology, Klinikum Forchheim, Forchheim, Germany; 3 Department of Gastroenterology, Klinikum Bayreuth, Bayreuth, Germany; 4 Institute of Translational Research, University of Birmingham, Birmingham, United Kingdom; 5 Faculty of Medicine, University Hospital Heidelberg, Ruprecht Karls University Heidelberg, Heidelberg, Germany; 6 Department of Medicine II, Division of Gastroenterology, Technical University Munich, Munich, Germany; 7 Gastroenterology & Digestive Endoscopy Unit, IRCCS Policlinico San Donato, San Donato Milanese, Milano, Italy; University Hospital Llandough, UNITED KINGDOM

## Abstract

**Background and aims:**

Dye-less chromoendoscopy is an emerging technology for colorectal polyp characterization. Herein, we investigated whether the newly introduced I-scan optical enhancement (OE) can accurately predict polyp histology *in vivo* in real-time.

**Methods:**

In this prospective three-phased study, 84 patients with 230 diminutive colorectal polyps were included. During the first two study phases, five endoscopists assessed whether analysis of polyp colour, surface and vascular pattern under i-scan OE can differentiate *in vivo* between adenomatous and hyperplastic polyps. Finally, junior and experienced endoscopists (JE, EE, each n = 4) not involved in the prior study phases made a post hoc diagnosis of polyp histology using a static i-scan OE image database. Histopathology was used as a gold-standard in all study phases.

**Results:**

The overall accuracy of i-scan OE for histology prediction was 90% with a sensitivity, specificity, positive (PPV) and negative prediction value (NPV) of 91%, 90%, 86% and 94%, respectively. In high confidence predictions, the diagnostic accuracy increased to 93% with sensitivity, specificity, PPV and NPV of 94%, 91%, 89% and 96%. Colonoscopy surveillance intervals were predicted correctly in ≥ 90% of patients. In the post hoc analysis EE predicted polyp histology under i-scan OE with an overall accuracy of 91%. After a single training session, JE achieved a comparable diagnostic performance for predicting polyp histology with i-scan OE.

**Conclusion:**

The histology of diminutive colorectal polyps can be accurately predicted with i-scan OE *in vivo* in real-time. Furthermore, polyp differentiation with i-scan OE appears to require only a short learning curve.

## Introduction

Colorectal cancer is one of the most common malignant disease prevalent in the population and has a high morbidity and mortality [1]. Colonoscopy has been established as a gold standard for colorectal cancer surveillance worldwide [2–4]. More than 50% of polyps detected during surveillance colonoscopy are diminutive polyps, i.e. ≤5mm in size [5] and at the same time, approximately half of these diminutive polyps are adenomas [6–13]. Since white light endoscopy cannot accurately differentiate between adenomatous and hyperplastic diminutive polyps [14, 15], it is standard to date to remove all polyps for subsequent histopathological analyses.

However, this redundant removal of all polyps is cost-, risk- and time-intensive and the annual up-front cost-savings in the US of forgoing pathology of diminutive polyps has been estimated to exceed 1 billion dollars per year [16]. Based on these considerations, the American Society of Gastrointestinal Endoscopy (ASGE) has proposed the so called PIVI (Preservation and Incorporation of Valuable endoscopic Innovations) statement [17] in which diagnostic thresholds are defined that new endoscopic techniques should meet in order to allow for valid and accurate *in vivo* prediction of polyp histology.

One of these technologies that holds the potential to fulfil the PIVI criteria is dye-less chromoendoscopy (DLC). Among the DLC-techniques, narrow band imaging (NBI) is one of the most investigated technologies. As shown in numerous studies, NBI can be utilized for precise and accurate tissue characterization [18, 19]. This profound evidence led to development of the NICE classification, which, by *in vivo* analyses of the colour, the surface pattern and the vascular pattern of diminutive polyps under NBI, allows to accurately differentiate adenomatous from hyperplastic polyps [20].

I-scan optical enhancement (OE) is a novel pre-processing optical chromoendoscopy technology that has recently been introduced to the market. As the physical principle of i-scan OE, the spectrum of the emitted light is reduced by optical filters to wavelengths that overlay with the absorption maximum of haemoglobin, thereby leading to an enhanced visualization of the mucosal and vascular pattern [21]. Since i-scan OE therefore follows a physical principle comparable to NBI, we hypothesized that (i) the analysis of polyp colour, surface and vasculature under i-scan OE can differentiate between adenomatous and hyperplastic polyps similar to the NICE classification for NBI, (ii) that polyp histology can be accurately predicted *in vivo* in real-time with i-scan even from investigators without prior experience in i-scan OE, (iii) that the characterization of polyp colour, surface and vascular pattern with i-scan OE as well as the differentiation between hyperplastic and adenomatous polyps with i-scan OE can be rapidly learned even from inexperienced endoscopists and addressed these issues in different phases in a prospective multicentre study.

## Materials and methods

### Patient recruitment and study setting

The study was designed as a three-phased prospective multicentre observational study conducted from January 2017 to June 2017 at the Ludwig Demling Endoscopy Center of Excellence at the University Hospital of Erlangen as well as three other academic educational hospitals. The study was approved by the Ethics committee of the Friedrich-Alexander University Erlangen (Krankenhausstrasse 12, 91054 Erlangen Germany) as well as the Institutional Review Board of the Medical Faculty of the Friedrich-Alexander University Erlangen-Nuremberg. No minors were included and written informed consent was obtained from all patients prior to the procedure. Patients with poor bowel preparation, colectomy, inflammatory bowel disease, anticoagulation or polyposis syndrome were excluded.

All colonoscopies were performed using a high-definition colonoscope equipped with the Pentax Medical OPTIVISTA EPK-i7010 video processor (Pentax, Tokyo, Japan). Diminutive polyps (≤5mm) identified during screening colonoscopy or colonoscopy initiated for the work-up of GI-related symptoms (bleeding, abdominal pain, change in bowel habits) were included. Four the purpose of our analysis, the boundary between the proximal and the distal colon was defined as the junction of the splenic flexure and the descending colon, as assessed by the endoscopist [22]. After visualization of the polyps in high definition white light (HDWL), the size (as compared to an open biopsy forceps with a diameter of 8 mm) and the location were recorded. Afterwards, i-scan OE was used to enhance the surface and vascular pattern and the endoscopist made a real-time *in vivo* prediction of the underlying histology based on colour, surface pattern and vessel pattern as described in the NICE classification [20]. Further, the endoscopist assigned a level of confidence (high or low) for the in vivo assessment, as previously described [19, 23, 24].

After optical assessment, polyps were resected using standard techniques and sent to an experienced GI pathologist for histological assessment. Finally, optical diagnosis was compared to histopathology as a reference standard. Diagnosis of the 7 SSAs included in the study, of which none exhibited cytologic dysplasia, was done by one experienced GI pathologist based on the WHO histopathological criteria.

### Study phases

**Fig 1:**
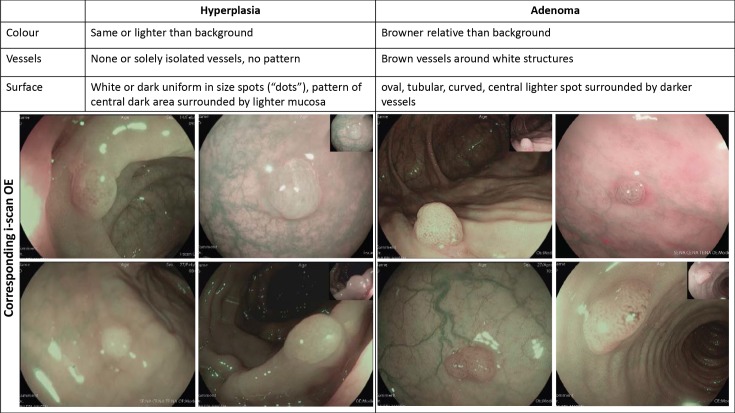
Endoscopic criteria under i-scan OE for *in vivo* assessment of polyp histology (adapted from [20]) and representative images.

During the first phase of the study, a single experienced investigator (>1000 colonoscopies performed) with expertise in optical diagnosis of polyp histology (T.R.) assessed whether polyp characterization according to colour, vascular and mucosal pattern as previously described for narrow band imaging [20], can similarly be used to distinguish between hyperplastic and adenomatous polyps *in vivo* using i-scan OE. For this validation phase, the first 50 polyps subsequently detected after study initiation were included. After an interims analysis showing that diminutive polyps can be well differentiated into hyperplastic and adenomatous polyps by i-scan OE *in vivo* with high accuracy using colour, surface pit pattern and vascular pattern as differentiating criteria (**Fig 1**), the study was extended to a total of five experienced endoscopists in its second phase in which these five different endoscopists similarly made an *in vivo* assessment of the polyp histology with i-scan OE in additional 61 patients. Polyps with endoscopic signs of malignancy as defined in the NICE classification [20] were not included in the study. In this second phase, the distinction between hyperplastic and adenomatous polyps was similarly made based on colour, surface pit pattern and vascular pattern and results were compared to histopathology as a reference standard. During the first and second phase, representative images of each polyp included were recorded and digitally stored as high-resolution images.

These images were then transferred into an anonymized consecutively numbered database. For this, only images in which the polyp was centered, sharp and clearly visible were considered, resulting in a total of 185 images of different polyps. This database was then used for the third phase of the study, in which junior endoscopists (n = 4) and experienced endoscopists (n = 4) not involved in the *in vivo* assessment during the first and second study phase made an optical diagnosis of the polyp histology based on the pictures in the database. Experienced endoscopist were defined as endoscopists with a specialty in gastroenterology and a vast number of endoscopies (>1000 colonoscopies) and experience in optical diagnosis and virtual chromoendoscopy. Junior endoscopists were defined as residents in gastroenterology who participated in less than 100 colonoscopies, never performed a colonoscopy independently and had no previous experience in optical or digital chromoendoscopy. Prior to image analysis from the database, junior endoscopists received a short training session of 30 minutes in which an expert in optical diagnosis (T.R.) explained the features of the NICE classification and how to distinguish adenomatous and hyperplastic polyps based on these criteria. The training session was conducted in a classroom environment with a PowerPoint presentation comprising of 17 representative NBI training static images used to explain the differentiation between Hyperplasia and Adenoma. After the training session and the actual post hoc image analysis by the junior endoscopists there was a time interval of 14 days. Optical diagnosis from the polyp image in the database images was done on a 15-inch laptop with a resolution of 1024 x 640 by each endoscopist individually in a single room without the possibility to interact with other endoscopists. No feedback was given during or after the optical diagnosis has been made.

### Statistics

The primary aim of this study was to assess the diagnostic accuracy including diagnostic sensitivity, specificity, positive (PPV) and negative prediction value (NPV) of i-scan OE for the in vivo prediction of polyp histology. The histopathology report was used as a reference for the validation of the endoscopic assessment. To assess the ability of i-scan OE to predict post-polypectomy surveillance intervals, the intervals that would be recommended by endoscopic prediction were compared with those that would be recommended by pathologic assessment as recommended in European [25] and US guidelines [26]. The probability for error (α) was set to 0.05 and the ß-error to 0.1 (reflecting a power of 0.90). For HDWL, an expected accuracy of 74% and for i-scan OE an expected accuracy of 90% was expected [24, 27, 28]. This resulted in a calculated sample size of at least 120 polyps to be included to sufficient statistical analysis.

## Results

### Patients and polyps characteristics

**Fig 2:**
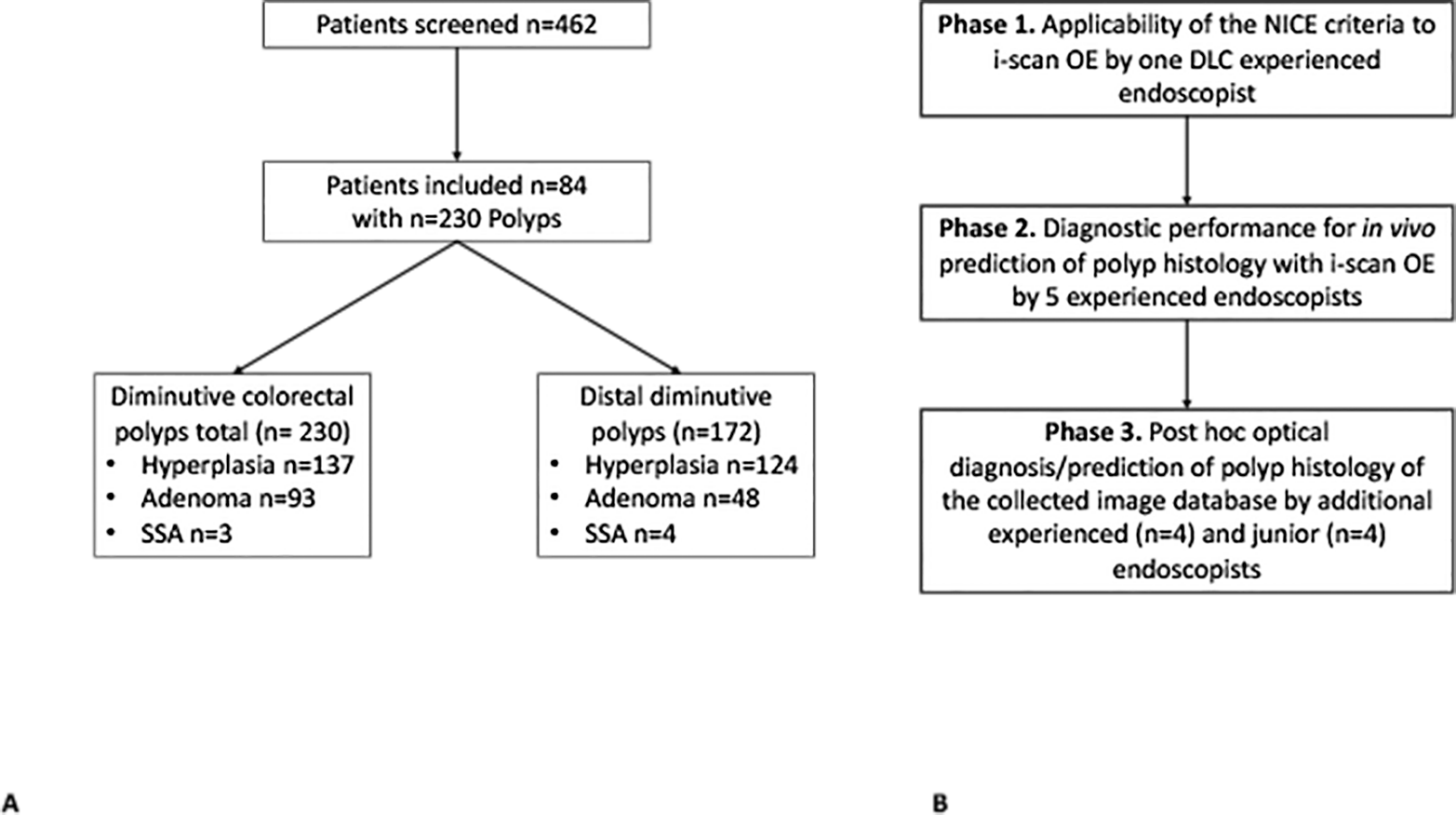
Patient recruitment and study phases. (A) Patient and polyp characteristics, SSA = sessile serrated adenoma (B) Exemplification of the study phases.

In total, 462 consecutive patients undergoing screening or surveillance colonoscopy were screened for study inclusion. Out of these, 84 patients exhibited a total of 230 diminutive colorectal polyps with an average size of 3.6 mm were included (**Fig 2A**). Of the 230 polyps, 58 were located proximal while 172 had a location in the descending colon (n = 12), sigmoid colon (n = 128) or rectum (n = 32) and hence represented distal polyps. Out of the proximal polyps, 9 were located in the caecum, 22 in the ascending colon and 27 in the transverse colon. Clinical and demographic patient characteristics and the clinical and histopathological characteristics of the 230 polyps are summarized in **Table 1**. In total, based on a common histopathological classification [29] 137 polyps were non-adenomatous, of which almost all were hyperplastic by histology (n = 136) while one was a granulomatous polyp. 40% of the polyps exhibited adenomatous histology (93 out of 230). Of these, the vast majority were tubular (72 out of 93) while 14 adenomas exhibited tubulovillous histology. Further, 7 adenomas were sessile serrated adenomas (SSA) on histology.

**Table 1:** Clinical and demographic characteristics of the patients and polyps.

	Patient characteristics	
	All patients	Patients with distal polyps only
Total, n	84	70
Sex, n (%)		
Male	53 (63)	46 (66)
Female	31 (37)	24 (34)
Age		
Mean ±SD	61±12	60±11
Median (range)	63 (25–88)	63 (25–88)
	**Polyp characteristics**	
Total, n	230	172
Location, n (%)		
Caecum	9 (4)	
Ascending colon	22 (10)	
Transverse colon	27 (12)	
Descending colon	12 (5)	12 (7)
Sigma	128 (56)	128 (74)
Rectum	32 (14)	32 (19)
**Histology, n (%)**		
Adenoma	93 (40)	48 (28)
Tubular	72 (31)	34 (20)
Tubulovillous	14 (6)	10 (6)
SSA	7 (3)	4 (2)
Hyperplastic	137 (60)	124 (72)
**Size, n (%)**		
≤3mm	115 (50)	97 (56)
4-5mm	115 (50)	75 (44)
Median (mean), mm	3 (4)	3 (4)

SD = standard deviation, SSA = sessile serrated adenoma

### Validation of the NICE classification for i-scan OE

For NBI the NICE classification has been established as an accurate and reliable method for polyp histology characterization [20]. Since i-scan OE follows a similar approach of reducing the emitted wavelengths by an optical filter to enhance surface and mucosal vascular pattern, we hypothesized that the visual characteristics of the NICE classification can be readily applied to i-scan OE for the *in vivo* differentiation of hyperplastic and adenomatous polyps. For this purpose, a total of 50 polyps were assessed by i-scan OE in the first phase of the study in which a single endoscopist experienced in optical diagnosis made an *in vivo* differentiation between hyperplasia and adenoma based on the criteria as set forth by the NICE classification (**Fig 2B**).

Indeed, when the previously for NBI validated criteria were applied to the first 50 polyps in this study, this allowed to accurately discriminate hyperplastic from adenomatous polyps. Specifically, the overall accuracy on prediction of colorectal polyp histology was 96% with a sensitivity, specificity, PPV and NPV of 100% (12/12, 95% CI, 70–100), 95% (36/38, 95% CI, 81–99), 86% (12/14, 95% CI, 56–98) and 100% (36/36, 95% CI, 88–100), respectively.

When only predictions made with high confidence were analysed, the overall accuracy increased to 100% and sensitivity, specificity, PPV and NPV were 100% (5/5, 95% CI, 46–100), 100% (32/32, 95% CI, 87–100), 100% (5/5, 95% CI, 46–100) and 100% (32/32, 95% CI, 87–100) for HC predictions, respectively. Since the “leave-in-place” strategy of the ASGE PIVI statement is focused on the management of diminutive polyps located in the rectosigmoid, a separate subgroup analysis for polyps in this location was performed. For polyps in the rectosigmoid, the overall diagnostic accuracy was 95%. Sensitivity, specificity, PPV and NPV were 100% (2/2, 95% CI, 20–100), 95% (39/41, 95% CI, 82–99), 50% (2/4, 95% CI, 9–91) and 100% (39/39, 95% CI, 89–100), respectively.

### *In vivo* real-time prediction of polyp histology by multiple expert endoscopists

After validation of the applicability of the NICE-classification to OE, the ongoing study was then extended in its second phase to a total of 5 experienced endoscopist, which made an optical diagnosis based on colour, surface and vascular pattern of further 180 diminutive colorectal polyps from 61 consecutively enrolled patients (**Fig 2B**).

In this second phase, the overall accuracy of histological prediction with i-scan OE was 90% with a sensitivity, specificity, PPV and NPV of 90% (73/81, 95% CI, 81–95), 88% (87/99, 95% CI, 79–93), 86% (73/85, 95% CI, 76–92) and 92% (87/95, 95% CI, 84–96), respectively. When only predictions made with high confidence were considered, diagnostic performances were markedly increased with a sensitivity of 94% (74/79, 95% CI, 85–98) and a specificity of 88% (74/84, 95% CI, 79–94). Likewise, positive and negative prediction were also increased to 88% (74/84, 95% CI, 79–94) and 94% (74/79, 95% CI, 85–98), respectively.

In distal diminutive colorectal polyps, the overall accuracy was 90%. Sensitivity, specificity, PPV and NPV were 94% (44/47, 95% CI, 8198), 88% (72/82, 95% CI, 78–94), 82% (44/54, 95% CI, 68–90) and 96% (72/75, 95% CI, 88–99), respectively. Since the second statement of the ASGE PIVI is focussed on polyps in the rectosigmoid, we performed subgroup analyses and found a sensitivity, specificity, PPV and NPV of 93% (26/28, 95% CI, 79–98), 90% (70/7895% CI, 83–9), 77% (26/34, 95% CI, 62–87) and 97% (70/72, 95% CI, 92–99) for polyps in the rectosigmoid only.

Combined diagnostic performances of i-scan OE made by the single investigator (phase 1) and the multiple investigator (phase 2) for the total of 230 polyps are summarized in **Table 2.**

**Table 2:** Diagnostic performances of i-scan OE for *in vivo* prediction of colorectal polyp histology during phase I and II of the study.

	Polyps (n)	Sensitivity (n,95% CI)	Specificity (n,95% CI)	PPV (n,95% CI)	NPV (n,95% CI)
**Single Investigator (SI, Phase I)**					
All polyps	50	100% (12/12, 70–100)	95% (36/38, 81–99)	86% (12/14, 56–98	100% (36/36, 88–100)
HC polyps	37	100% (5/5, 46–100)	100% (32/32, 87–100)	100% (5/5, 46–100)	100% (32/32, 87–100)
Distal polyps	43	100% (2/2, 19.8–100)	95% (39/41, 82–99)	50% (2/4, 9–91)	100% (39/39, 89–100)
**Multiple investigators (MI, Phase II)**					
All polyps	180	90% (73/81, 81–95)	88% (87/99, 79–93)	86% (73/85, 76–92	92% (87/95, 84–96)
HC polyps	163	94% (74/79, 85–98)	88% (74/84, 79–94)	88% (74/84, 79–94)	94% (74/79, 85–98)
Distal polyps	129	94% (44/47, 81–98)	88% (72/82, 78–94)	82% (44/54, 68–90)	96% (72/75, 88–99)
**SI+MI (Phase I + II)**					
All polyps	230	91% (85/93, 83–96)	90% (123/137, 83–94)	86% (85/99, 77–92)	94% (123/131, 88–97)
HC polyps	200	94% (79/84, 86–98)	91% (106/116, 84–96)	89% (79/89, 80–94)	96% (106/111, 89–98)
Distal polyps	172	94% (46/49, 82–98)	90% (111/123, 83–95)	79% (46/58, 66–88)	97% (111/114, 92–99.)

HC = high confidence, CI = confidence interval

#### Prediction of the colonoscopy surveillance intervals

To assess the ability to predict post-polypectomy surveillance intervals by i-scan OE, the intervals that would be recommended by prediction with optical *in vivo* assessment system were compared with those that would be recommended by pathologic assessment. For the later, European [25] and US [26] guidelines were used to determine post-polypectomy surveillance intervals. In accordance with the resect and discard paradigm of the ASGE PIVI, only polyps with HC prediction were included in these analyses and determination of post-polypectomy surveillance was made on a per-patient level. With i-scan OE a correct prediction of surveillance interval was possible in 82 out of 84 patients (98%) based on European guidelines, whereas 80 out of 84 (95%) were predicted correctly according to the US guidelines.

When only distal colorectal polyps were considered, surveillance intervals based on US guidelines were predicted correctly in 67 out of the 70 patients (96%) whilst surveillance intervals were in agreement in 69 of 70 patients (99%) with distal colorectal polyps when surveillance was recommend based on European guidelines. Importantly, in all cases where the colonoscopy intervals as determined by i-scan OE were not predicted correctly, in both European and US guidelines, the i-scan OE intervals would have been shorter than the histopathologically guided intervals. Patients with differences in the colonoscopy surveillance interval determined by i-scan OE and by histopathology are shown in **Table 3**.

**Table 3:** Patients with differences between histopathological and i-scan OE surveillance-colonoscopy-recommendations.

	Surveillance by Histology (years)		Surveillance by i-scan OE (years)		Most advanced pathology
	EU	US	EU	US	
**All polyps**					
Patient 1	10	10	10	5	hyperplastic
Patient 2	10	5	3	3	1 tubular adenoma
Patient 3	10	10	10	5	hyperplastic
Patient 4	10	5	3	3	2 tubular adenomas
**Distal Polyps**					
Patient 1	10	10	10	5	hyperplastic
Patient 2	10	5	3	3	1 tubular adenoma
Patient 3	10	10	10	5	hyperplastic

### Histology prediction with i-scan OE by junior endoscopists and experienced endoscopists

In the last phase of the study, the prediction of polyp histology with i-scan OE by junior endoscopist and experienced endoscopists not involved in the prior conducted endoscopies was assessed on the image database of a total of 185 images of different polyps collected during the first and second phase of the study.

For experienced endoscopists, all of whom had prior experience in optical and digital chromoendoscopy, the overall accuracy was 91%, with average sensitivity and specificity of 87% and 92%, respectively.

After only one single training session, junior endoscopists correctly assessed polyp histology with i-scan OE with an overall accuracy of 89% with average sensitivity and specificity of 89% and 90%, respectively and thereby achieved comparable results to the expert endoscopists.

A summary of the individual diagnostic performances of junior and experienced endoscopists is shown in **Table 4.**

**Table 4:** Diagnostic performances of junior and experienced endoscopists with i-scan OE during phase III of the study.

	Junior endoscopists (JE, n = 4)				Experienced endoscopists (EE, n = 4)			
	JE 1	JE 2	JE 3	JE 4	EE 1	EE 2	EE 3	EE 4
**Sensitivity (n, 95% CI)**	87%, (62/71, 77–94)	86%, (61/71, 75–93)	92%, (65/71, 82–97)	93%, (66/71, 84–97)	87%, (62/71, 77–94)	79%, (56/71, 67–87)	93%, (66/71, 84–97)	89%, (63/71, 79–95)
**Specificity (n, 95% CI)**	85%, (97/114, 77–91)	91%, (104/114, 84–96)	91%, (104/114, 84–96)	90%, (103/114, 83–95)	88%, (100/114, 80–93)	97%, (111/114, 92–99)	90%, (103/114, 83–95)	94%, (107/1114, 87–97)
**PPV** **(n, 95% CI)**	79%, (62/79, 68–87)	86%, (61/71, 75–93)	87%, (65/75, 76–93)	86%, (66/77, 76–92)	82%, (62/76, 71–89)	95%, (56/59, 85–99)	86%, (66/77, 76–92)	90%, (63/70, 80–96)
**NPV** **(n, 95% CI)**	92%, (97/106, 84–96)	91%, (104/114, 84–96)	95%, (104/110, 88–98)	95%, (103/108, 89–98)	92%, (100/109, 85–96)	88%, (111/126, 81–93)	95%, (103/108, 89–98)	93%, (107/115, 86–97)

## Discussion

In this study we successfully showed for the first time that: 1) the NICE classification for the differentiation of adenomatous and hyperplastic polyps is applicable to i-scan OE, 2) i-scan OE meets the criteria of the ASGE PIVI statement for resecting and discarding diminutive polyps without histological assessment and for leaving distal diminutive colorectal polyps in place and 3) differentiation between hyperplastic and adenomatous polyps with i-scan OE appears to be easily learnable and can be readily utilized even by junior endoscopists after a single training session.

Among the various technologies for dye-less chromoendoscopy (such as NBI, i-scan, FICE), NBI is amidst the most intensively studied for optical diagnosis of polyp histology. Apart from demonstrating reliable endoscopic characterisation and differentiation of hyperplastic and adenomatous polyps, conformity of NBI to the thresholds as set forth in the ASGE PIVI statement has been shown in numerous studies [18, 19, 30].

This profound evidence on the reliability of NBI for the characterization of colorectal polyps culminated in a NBI-based classification system for diminutive colorectal polyps [20]: as shown in 2012 by Douglas Rex and co-workers, the evaluation of polyp colour, surface pattern and vessel pattern under NBI allowed to precisely and reliably distinguish between hyperplastic and adenomatous polyps.

I-scan optical enhancement was introduced to the market in 2016 and since the optical enhancement technology follows a similar principle of reducing the spectral bandwidth of the emitted light by an optical filter to wavelengths that correspond to the peak absorbance maximum of haemoglobin, we hypothesized that the originally for NBI developed NICE classification can well be utilized for i-scan OE to predict the histology of diminutive colorectal polyps.

As shown by our results, i-scan OE by far exceeded the recommended threshold criteria of the ASGE PIVI statement. Specifically, the negative prediction for ruling out adenomatous histology was 97% in rectosigmoid polyps in our study and hence, i-scan OE appears to allow leaving distal polyps in place without resecting them as proposed by the ASGE. In addition to that, our results of a very high negative predictive value in optical assessment of polyps throughout the entire colon indicate that the leave in place strategy might not only be limited to polyps in the rectosigmoid, but might also be applicable for polyps in the entire colon. Furthermore, the overall accuracy of post-polypectomy surveillance colonoscopy prediction with i-scan OE was 98.6% when using EU and 95.7% when using US guideline criteria and hence also exceeded the threshold criteria for the resect and discard strategy. Importantly, in those patients where the surveillance interval was predicted incorrectly, the intervals as recommended by optical diagnosis with i-scan OE would have been shorter compared to histopathology based surveillance. This overestimation is a direct reflection of the higher sensitivity of i-scan OE for histology assessment compared to its specificity and it can therefore be reasoned that no potential adenoma would have been missed, since the patient would have come sooner to surveillance colonoscopy.

Although i-scan OE shows a promising potential in the characterization of colorectal polyps, the direct clinical applicability has yet to be further evaluated in larger studies. Specifically, it is not clear whether optical diagnosis with i-scan OE results indeed in actual cost savings. In this regard, it might be speculated that the cost savings which are conferred by optical diagnosis and associated reduced pathology costs are counterbalanced by the overestimation of polyp histology (i.e. that hyperplastic polyps are predicted to exhibit adenomatous histology) with i-scan OE and the subsequently narrower colonoscopy intervals that is associated with this overestimation when surveillance is based on optical diagnosis. It can be argued that it might be better from an individual patient's standpoint to have every polyp found also reviewed by a pathologist to ensure they are screened at an appropriate interval rather than following a narrower colonoscopic surveillance. In this regard, it has also be taken into account that patient acceptance of optical diagnosis still appears to be limited [31, 32].

Since approximately 20 to 30% of colorectal cancers arise through the serrated pathway [33, 34], the reliable endoscopic identification of SSAs is of great importance. However, as shown in a recent meta-analysis, optical diagnosis with image enhanced endoscopy exhibited only a pooled sensitivity for discriminating SSAs from non-neoplastic lesion of 80% [35]. Although limited in number and thereby hindering far reaching conclusions, of the 7 SSAs included in the study only 5 were correctly predicted be to adenomatous lesions by i-scan OE. Based on this, larger studies that assess whether i-scan OE can accurately predict histology in SSAs are highly warranted.

In order for an endoscopic technique to be introduced into clinical practice it has to be easily learned. Therefore, in the last part of the study we assessed whether junior endoscopists with little or no experience in endoscopy could discriminate hyperplastic against adenomatous polyps with i-scan OE and compared the ability to predict polyp histology with that of experienced endoscopists using a static database of i-scan OE images. As expected, the experienced endoscopists were directly able to predict the histology with high overall accuracy when i-scan OE images were shown. Remarkably, however, after a short didactic training session of 30 minutes and a “wash-out phase” of 14 day, also junior endoscopists were able to predict polyp histology with a diagnostic accuracy comparable to experienced endoscopists. Therefore, assessment of polyp histology with i-scan OE appears to require only a short learning, an observation which is very consistent with data on the learning curve of other DLC technologies [36–39].

These results might also have direct implications to the evolving field of molecular pathological epidemiology (MPE) as an integrative molecular and population health based approach to address molecular pathogenesis and heterogeneity of disease processes [40, 41]. In this regard, MPE of colon and rectal premalignant lesions together with optical diagnosis might offer the unique opportunity to identify a certain risk-profile for developing colorectal neoplasia and by subclassifying disease of interest by *in vivo* pathologic features, this can contribute to biomarker research and precision medicine [42].

Potential limitations should also be discussed; one of which is the fact that during the first two study phases, only experienced endoscopists used i-scan OE for histology prediction. Although our results in the third phase indicate that accurate optical diagnosis of polyp histology can be accomplished by inexperienced endoscopists, it is still clear that our findings of the accurate assessment of polyp histology during ongoing endoscopy needs to be verified in separate studies involving also non-tertiary referral centres or non-teaching hospitals as well as gastroenterologists in private practice. This aspect seems to be of certain importance since a recent study with i-scan (without optical enhancement) including 10 private practice endoscopists found that the endoscopic classification of polyp type is not accurate enough to abandon histopathology [43]. Further, a washout phase of 14 days might have been too short and hence might have directly influenced the decision making process when attempting an optical diagnosis by the junior endoscopists.

In the future, additional multi-centre studies including comparative analysis of optical diagnosis with i-scan OE between expert and non-expert endoscopists will be necessary before this approach can be implemented into daily endoscopic routine.

## References

[1] Baxter NN, Goldwasser MA, Paszat LF, Saskin R, Urbach DR, Rabeneck L. Association of colonoscopy and death from colorectal cancer. Ann Intern Med. 2009;150(1):1–8. . 19075198 10.7326/0003-4819-150-1-200901060-00306

[2] Schoenfeld P, Cash B, Flood A, Dobhan R, Eastone J, Coyle W, et al Colonoscopic screening of average-risk women for colorectal neoplasia. N Engl J Med. 2005;352(20):2061–8. doi: 10.1056/NEJMoa042990 . 15901859 10.1056/NEJMoa042990

[3] Detsky AS. Screening for colon cancer—can we afford colonoscopy? N Engl J Med. 2001;345(8):607–8. doi: 10.1056/NEJM200108233450809 . 11529216 10.1056/NEJM200108233450809

[4] Levin B, Lieberman DA, McFarland B, Andrews KS, Brooks D, Bond J, et al Screening and surveillance for the early detection of colorectal cancer and adenomatous polyps, 2008: a joint guideline from the American Cancer Society, the US Multi-Society Task Force on Colorectal Cancer, and the American College of Radiology. Gastroenterology. 2008;134(5):1570–95. doi: 10.1053/j.gastro.2008.02.002 . 18384785 10.1053/j.gastro.2008.02.002

[5] Rex DK, Helbig CC. High yields of small and flat adenomas with high-definition colonoscopes using either white light or narrow band imaging. Gastroenterology. 2007;133(1):42–7. doi: 10.1053/j.gastro.2007.04.029 . 17631129 10.1053/j.gastro.2007.04.029

[6] Lieberman D, Moravec M, Holub J, Michaels L, Eisen G. Polyp size and advanced histology in patients undergoing colonoscopy screening: implications for CT colonography. Gastroenterology. 2008;135(4):1100–5. doi: 10.1053/j.gastro.2008.06.083 ; PubMed Central PMCID: PMCPMC2581902. 18691580 10.1053/j.gastro.2008.06.083 PMC2581902

[7] Butterly LF, Chase MP, Pohl H, Fiarman GS. Prevalence of clinically important histology in small adenomas. Clin Gastroenterol Hepatol. 2006;4(3):343–8. doi: 10.1016/j.cgh.2005.12.021 . 16527698 10.1016/j.cgh.2005.12.021

[8] Schoen RE, Hur C. What is the clinical importance of small polyps with regard to colorectal cancer screening? Nat Clin Pract Gastroenterol Hepatol. 2006;3(9):488–9. doi: 10.1038/ncpgasthep0599 . 16951664 10.1038/ncpgasthep0599

[9] Morson B. President's address. The polyp-cancer sequence in the large bowel. Proc R Soc Med. 1974;67(6 Pt 1):451–7. ; PubMed Central PMCID: PMCPMC1645739. 4853754 10.1177/00359157740676P115 PMC1645739

[10] Shinya H, Wolff WI. Morphology, anatomic distribution and cancer potential of colonic polyps. Ann Surg. 1979;190(6):679–83. ; PubMed Central PMCID: PMCPMC1345622. 518167 10.1097/00000658-197912000-00001 PMC1345622

[11] Matek W, Guggenmoos-Holzmann I, Demling L. Follow-up of patients with colorectal adenomas. Endoscopy. 1985;17(5):175–81. doi: 10.1055/s-2007-1018494 . 4054062 10.1055/s-2007-1018494

[12] Church JM. Clinical significance of small colorectal polyps. Dis Colon Rectum. 2004;47(4):481–5. doi: 10.1007/s10350-003-0078-6 . 14994108 10.1007/s10350-003-0078-6

[13] Rex DK, Overhiser AJ, Chen SC, Cummings OW, Ulbright TM. Estimation of impact of American College of Radiology recommendations on CT colonography reporting for resection of high-risk adenoma findings. Am J Gastroenterol. 2009;104(1):149–53. doi: 10.1038/ajg.2008.35 . 19098863 10.1038/ajg.2008.35

[14] Hoffman A, Sar F, Goetz M, Tresch A, Mudter J, Biesterfeld S, et al High definition colonoscopy combined with i-Scan is superior in the detection of colorectal neoplasias compared with standard video colonoscopy: a prospective randomized controlled trial. Endoscopy. 2010;42(10):827–33. doi: 10.1055/s-0030-1255713 . 20803419 10.1055/s-0030-1255713

[15] Leufkens AM, van Oijen MG, Vleggaar FP, Siersema PD. Factors influencing the miss rate of polyps in a back-to-back colonoscopy study. Endoscopy. 2012;44(5):470–5. doi: 10.1055/s-0031-1291666 . 22441756 10.1055/s-0031-1291666

[16] Kessler WR, Imperiale TF, Klein RW, Wielage RC, Rex DK. A quantitative assessment of the risks and cost savings of forgoing histologic examination of diminutive polyps. Endoscopy. 2011;43(8):683–91. doi: 10.1055/s-0030-1256381 . 21623556 10.1055/s-0030-1256381

[17] Rex DK, Kahi C, O'Brien M, Levin TR, Pohl H, Rastogi A, et al The American Society for Gastrointestinal Endoscopy PIVI (Preservation and Incorporation of Valuable Endoscopic Innovations) on real-time endoscopic assessment of the histology of diminutive colorectal polyps. Gastrointest Endosc. 2011;73(3):419–22. doi: 10.1016/j.gie.2011.01.023 . 21353837 10.1016/j.gie.2011.01.023

[18] Gupta N, Bansal A, Rao D, Early DS, Jonnalagadda S, Edmundowicz SA, et al Accuracy of in vivo optical diagnosis of colon polyp histology by narrow-band imaging in predicting colonoscopy surveillance intervals. Gastrointest Endosc. 2012;75(3):494–502. doi: 10.1016/j.gie.2011.08.002 . 22032847 10.1016/j.gie.2011.08.002

[19] Rex DK. Narrow-band imaging without optical magnification for histologic analysis of colorectal polyps. Gastroenterology. 2009;136(4):1174–81. doi: 10.1053/j.gastro.2008.12.009 . 19187781 10.1053/j.gastro.2008.12.009

[20] Hewett DG, Kaltenbach T, Sano Y, Tanaka S, Saunders BP, Ponchon T, et al Validation of a simple classification system for endoscopic diagnosis of small colorectal polyps using narrow-band imaging. Gastroenterology. 2012;143(3):599–607 e1. doi: 10.1053/j.gastro.2012.05.006 . 22609383 10.1053/j.gastro.2012.05.006

[21] Iacucci M, Kiesslich R, Gui X, Panaccione R, Heatherington J, Akinola O, et al Beyond white light: optical enhancement in conjunction with magnification colonoscopy for the assessment of mucosal healing in ulcerative colitis. Endoscopy. 2017;49(6):553–9. doi: 10.1055/s-0042-124363 . 28315280 10.1055/s-0042-124363

[22] Imperiale TF, Wagner DR, Lin CY, Larkin GN, Rogge JD, Ransohoff DF. Risk of advanced proximal neoplasms in asymptomatic adults according to the distal colorectal findings. N Engl J Med. 2000;343(3):169–74. doi: 10.1056/NEJM200007203430302 . 10900275 10.1056/NEJM200007203430302

[23] Hewett DG, Huffman ME, Rex DK. Leaving distal colorectal hyperplastic polyps in place can be achieved with high accuracy by using narrow-band imaging: an observational study. Gastrointest Endosc. 2012;76(2):374–80. doi: 10.1016/j.gie.2012.04.446 . 22695207 10.1016/j.gie.2012.04.446

[24] Basford PJ, Longcroft-Wheaton G, Higgins B, Bhandari P. High-definition endoscopy with i-Scan for evaluation of small colon polyps: the HiSCOPE study. Gastrointest Endosc. 2014;79(1):111–8. doi: 10.1016/j.gie.2013.06.013 . 23871094 10.1016/j.gie.2013.06.013

[25] Atkin WS, Valori R, Kuipers EJ, Hoff G, Senore C, Segnan N, et al European guidelines for quality assurance in colorectal cancer screening and diagnosis. First Edition—Colonoscopic surveillance following adenoma removal. Endoscopy. 2012;44 Suppl 3:SE151–63. doi: 10.1055/s-0032-1309821 . 23012119 10.1055/s-0032-1309821

[26] Lieberman DA, Rex DK, Winawer SJ, Giardiello FM, Johnson DA, Levin TR. Guidelines for colonoscopy surveillance after screening and polypectomy: a consensus update by the US Multi-Society Task Force on Colorectal Cancer. Gastroenterology. 2012;143(3):844–57. doi: 10.1053/j.gastro.2012.06.001 . 22763141 10.1053/j.gastro.2012.06.001

[27] Lee CK, Lee SH, Hwangbo Y. Narrow-band imaging versus I-Scan for the real-time histological prediction of diminutive colonic polyps: a prospective comparative study by using the simple unified endoscopic classification. Gastrointest Endosc. 2011;74(3):603–9. doi: 10.1016/j.gie.2011.04.049 . 21762907 10.1016/j.gie.2011.04.049

[28] Rastogi A, Early DS, Gupta N, Bansal A, Singh V, Ansstas M, et al Randomized, controlled trial of standard-definition white-light, high-definition white-light, and narrow-band imaging colonoscopy for the detection of colon polyps and prediction of polyp histology. Gastrointest Endosc. 2011;74(3):593–602. doi: 10.1016/j.gie.2011.04.050 . 21802078 10.1016/j.gie.2011.04.050

[29] Lambert R, Kudo SE, Vieth M, Allen JI, Fujii H, Fujii T, et al Pragmatic classification of superficial neoplastic colorectal lesions. Gastrointest Endosc. 2009;70(6):1182–99. doi: 10.1016/j.gie.2009.09.015 . 19879563 10.1016/j.gie.2009.09.015

[30] Rastogi A, Keighley J, Singh V, Callahan P, Bansal A, Wani S, et al High accuracy of narrow band imaging without magnification for the real-time characterization of polyp histology and its comparison with high-definition white light colonoscopy: a prospective study. Am J Gastroenterol. 2009;104(10):2422–30. doi: 10.1038/ajg.2009.403 . 19584829 10.1038/ajg.2009.403

[31] Sakata S, Lee AHS, Kheir AO, Tutticci NJ, Naidu S, Stevenson ARL, et al Patient acceptance of the optical diagnosis and misdiagnosis of diminutive colorectal polyps. Gastrointest Endosc. 2017;86(2):372–5 e2. doi: 10.1016/j.gie.2016.11.031 . 27931950 10.1016/j.gie.2016.11.031

[32] Rex DK, Patel NJ, Vemulapalli KC. A survey of patient acceptance of resect and discard for diminutive polyps. Gastrointest Endosc. 2015;82(2):376–80 e1. doi: 10.1016/j.gie.2015.04.029 . 26071067 10.1016/j.gie.2015.04.029

[33] Rex DK, Ahnen DJ, Baron JA, Batts KP, Burke CA, Burt RW, et al Serrated lesions of the colorectum: review and recommendations from an expert panel. Am J Gastroenterol. 2012;107(9):1315–29; quiz 4, 30. doi: 10.1038/ajg.2012.161 ; PubMed Central PMCID: PMCPMC3629844. 22710576 10.1038/ajg.2012.161 PMC3629844

[34] Erichsen R, Baron JA, Hamilton-Dutoit SJ, Snover DC, Torlakovic EE, Pedersen L, et al Increased Risk of Colorectal Cancer Development Among Patients With Serrated Polyps. Gastroenterology. 2016;150(4):895–902 e5. doi: 10.1053/j.gastro.2015.11.046 . 26677986 10.1053/j.gastro.2015.11.046

[35] Parikh ND, Chaptini L, Njei B, Laine L. Diagnosis of sessile serrated adenomas/polyps with image-enhanced endoscopy: a systematic review and meta-analysis. Endoscopy. 2016;48(8):731–9. doi: 10.1055/s-0042-107592 . 27223636 10.1055/s-0042-107592

[36] Neumann H, Vieth M, Fry LC, Gunther C, Atreya R, Neurath MF, et al Learning curve of virtual chromoendoscopy for the prediction of hyperplastic and adenomatous colorectal lesions: a prospective 2-center study. Gastrointest Endosc. 2013;78(1):115–20. doi: 10.1016/j.gie.2013.02.001 . 23528656 10.1016/j.gie.2013.02.001

[37] Bouwens MW, de Ridder R, Masclee AA, Driessen A, Riedl RG, Winkens B, et al Optical diagnosis of colorectal polyps using high-definition i-scan: an educational experience. World J Gastroenterol. 2013;19(27):4334–43. doi: 10.3748/wjg.v19.i27.4334 ; PubMed Central PMCID: PMCPMC3718901. 23885144 10.3748/wjg.v19.i27.4334 PMC3718901

[38] Ignjatovic A, Thomas-Gibson S, East JE, Haycock A, Bassett P, Bhandari P, et al Development and validation of a training module on the use of narrow-band imaging in differentiation of small adenomas from hyperplastic colorectal polyps. Gastrointest Endosc. 2011;73(1):128–33. doi: 10.1016/j.gie.2010.09.021 . 21184878 10.1016/j.gie.2010.09.021

[39] Raghavendra M, Hewett DG, Rex DK. Differentiating adenomas from hyperplastic colorectal polyps: narrow-band imaging can be learned in 20 minutes. Gastrointest Endosc. 2010;72(3):572–6. doi: 10.1016/j.gie.2010.03.1124 . 20561618 10.1016/j.gie.2010.03.1124

[40] Lochhead P, Chan AT, Giovannucci E, Fuchs CS, Wu K, Nishihara R, et al Progress and opportunities in molecular pathological epidemiology of colorectal premalignant lesions. Am J Gastroenterol. 2014;109(8):1205–14. doi: 10.1038/ajg.2014.153 ; PubMed Central PMCID: PMCPMC4125459. 24935274 10.1038/ajg.2014.153 PMC4125459

[41] Ogino S, Nishihara R, VanderWeele TJ, Wang M, Nishi A, Lochhead P, et al Review Article: The Role of Molecular Pathological Epidemiology in the Study of Neoplastic and Non-neoplastic Diseases in the Era of Precision Medicine. Epidemiology. 2016;27(4):602–11. doi: 10.1097/EDE.0000000000000471 ; PubMed Central PMCID: PMCPMC4892980. 26928707 10.1097/EDE.0000000000000471 PMC4892980

[42] Ogino S, Chan AT, Fuchs CS, Giovannucci E. Molecular pathological epidemiology of colorectal neoplasia: an emerging transdisciplinary and interdisciplinary field. Gut. 2011;60(3):397–411. doi: 10.1136/gut.2010.217182 ; PubMed Central PMCID: PMCPMC3040598. 21036793 10.1136/gut.2010.217182 PMC3040598

[43] Schachschal G, Mayr M, Treszl A, Balzer K, Wegscheider K, Aschenbeck J, et al Endoscopic versus histological characterisation of polyps during screening colonoscopy. Gut. 2014;63(3):458–65. doi: 10.1136/gutjnl-2013-304562 . 23812324 10.1136/gutjnl-2013-304562

